# Characterization of the Complete Mitochondrial Genome of Basidiomycete Yeast *Hannaella oryzae*: Intron Evolution, Gene Rearrangement, and Its Phylogeny

**DOI:** 10.3389/fmicb.2021.646567

**Published:** 2021-05-28

**Authors:** Qiang Li, Lijiao Li, Huiyu Feng, Wenying Tu, Zhijie Bao, Chuan Xiong, Xu Wang, Yuan Qing, Wenli Huang

**Affiliations:** ^1^Key Laboratory of Coarse Cereal Processing, Ministry of Agriculture and Rural Affairs, School of Food and Biological Engineering, Chengdu University, Chengdu, China; ^2^Biotechnology and Nuclear Technology Research Institute, Sichuan Academy of Agricultural Sciences, Chengdu, China; ^3^College of Life Sciences, Henan Agricultural University, Zhengzhou, China; ^4^Panxi Featured Crops Research and Utilization Key Laboratory of Sichuan Province, Xichang University, Xichang, China

**Keywords:** *Hannaella*, mitochondrial genome, protein coding gene, repeat sequence, gene rearrangement, phylogenetic analysis 3

## Abstract

In this study, the mitogenome of *Hannaella oryzae* was sequenced by next-generation sequencing (NGS) and successfully assembled. The *H. oryzae* mitogenome comprised circular DNA molecules with a total size of 26,444 bp. We found that the mitogenome of *H. oryzae* partially deleted the tRNA gene transferring cysteine. Comparative mitogenomic analyses showed that intronic regions were the main factors contributing to the size variations of mitogenomes in Tremellales. Introns of the *cox1* gene in Tremellales species were found to have undergone intron loss/gain events, and introns of the *H. oryzae cox1* gene may have different origins. Gene arrangement analysis revealed that *H. oryzae* contained a unique gene order different from other Tremellales species. Phylogenetic analysis based on a combined mitochondrial gene set resulted in identical and well-supported topologies, wherein *H. oryzae* was closely related to *Tremella fuciformis*. This study represents the first report of mitogenome for the *Hannaella* genus, which will allow further study of the population genetics, taxonomy, and evolutionary biology of this important phylloplane yeast and other related species.

## Introduction

*Hannaella* is a basidiomycetous yeast genus belonging to the order Tremellales, phylum Basidiomycota. It was proposed to accommodate species closely related to the genera *Derxomyces* and *Dioszegia*, which belong to the *Bullera sinensis* clade of the *Luteolus* lineage of the Tremellales based on a series of molecular markers (Wang and Bai, 2008; Kaewwichian et al., 2015; Han et al., 2017). So far, about 12 species have been described in this genus, including *Hannaella coprosmaensis*, *Hannaella dianchiensis*, *Hannaella kunmingensis*, *Hannaella luteola*, *Hannaella phyllophila*, *Hannaella phetchabunensis*, *Hannaella pagnoccae*, *Hannaella surugaensis*, *Hannaella sinensis*, *Hannaella siamensis*, *Hannaella zeae*, and *Hannaella oryzae* (Landell et al., 2014; Kaewwichian et al., 2015; Han et al., 2017). These species are found widely distributed on the leaf surfaces of various plants, including rice, wheat, and fruit trees (Han et al., 2017). As an important phyllosphere-inhabiting yeast, *H. oryzae* was considered to play an important role in promoting plant growth and biocontrol. This genus is considered to be monophyletic in nature, and *H. kunmingensis* showed genotypic and phenotypic variability (Dayo-Owoyemi et al., 2013). Both the basidiomycetous and the ascomycetous yeasts have been found colonizing on phylloplane from various regions of the world (Glushakova et al., 2007; Landell et al., 2010; Molnarova et al., 2014). These basidiomycetous genera were found to be the most common phylloplane yeasts, including *Sporobolomyces*, *Rhodotorula*, *Cryptococcus*, *Trichosporon*, and *Hannaella* (de Azeredo et al., 1998; Kaewwichian et al., 2015). The mitochondrial genomic characteristics of representative species of the two phylloplane yeast genera (*Cryptococcus* and *Trichosporon*) have been published, which facilitated our understanding of phylloplane yeasts (Yang et al., 2012; Gomes et al., 2018; Yan et al., 2018). However, no mitochondrial genome has been published on the genus *Hannaella* or the family Bulleribasidiaceae. *H. oryzae* has been isolated from the phylloplane of various plants. The report of its mitochondrial genome will help us understand the genetic characteristics of this important phylloplane yeast.

Mitochondrial genomes are effective tools for analyzing the phylogenetic and genetic evolution of eukaryotes because they contain many available molecular markers (Burger et al., 2003; Bullerwell and Lang, 2005; Cheng et al., 2021). Besides, the arrangement of mitochondrial genes, their transfer RNA (tRNA) structure, their codon usage, and the dynamic changes of introns can also be used to infer the evolutionary status of eukaryotes (Li et al., 2018a; Wang et al., 2018, 2020b; Wu et al., 2021). With the rapid development of next-generation sequencing technologies in recent years, more and more mitochondrial genomes have been obtained (du Toit et al., 2017; Yang et al., 2018; Zhang et al., 2018; Wang et al., 2020c). However, the mitochondrial genomes of fungi are less studied than those of animals. So far, less than 130 basidiomycete mitochondrial genomes have been reported^1^, which limits our understanding of the “second genome” (mitochondrial genome) of fungi. Studies on the fungal mitogenomes have shown that fungal mitogenomes exhibited significant variations in gene order, introns, intergenic regions, genome size, and repetitive sequences (Zhang et al., 2016; Zhang S. et al., 2017; Zhang Y.J. et al., 2017; Fourie et al., 2018; Wang et al., 2020a; Li et al., 2021). Despite enormous variations in the fungal genome, the 15 protein-coding genes, including *atp6*, *atp8*, *atp9*, *cob*, *cox1*, *cox2*, *cox3*, *nad1*, *nad2*, *nad3*, *nad4*, *nad4L*, *nad5*, *nad6*, and *rps3*, have been detected in most basidiomycete mitochondrial genomes, which were considered to be core protein-coding genes (PCGs) in the basidiomycete mitochondrial genomes.

In the present study, the complete mitochondrial genome of *H. oryzae* was sequenced and assembled by next-generation sequencing technology. The content, organization, and structure of the mitochondrial genes were revealed. We compared the mitochondrial genome of *H. oryzae* with its closely related species to identify variations and similarities in the gene content, genome organization, and gene order. The dynamic changes of introns were also revealed in *H. oryzae* and other basidiomycete species. In addition, the phylogenetic relationships among various basidiomycete species based on combined mitochondrial gene sets were analyzed. The mitochondrial genome of *H. oryzae* will allow further study of the population genetics, taxonomy, and evolutionary biology of this important phylloplane yeast and other related species.

## Materials and Methods

### Sample Collection, DNA Extraction, and Sequencing

The *H. oryzae* strain s11 was isolated from the surface of corn leaves collected in Sichuan, China, using the improved ballistoconidia-fall method as described by Kaewwichian et al. (2015). The morphological, biochemical, and physiological characteristics of the collected yeast strains were examined according to standard methods described by Kurtzman et al. (2011). The total genomic DNA was extracted using the method described by Wang and Bai (2008). *H. oryzae* was further identified based on the internal transcribed spacer (ITS) sequence and the mitochondrial *cob* gene. Whole-genome sequencing libraries were constructed using NEBNext Ultra II DNA Library Prep Kits (NEB, Beijing, China) with the extracted genomic DNA following the manufacturer’s instructions. Whole-genome sequencing was carried out on an Illumina HiSeq 2500 Platform (Illumina, San Diego, CA, United States). To verify the accuracy and integrity of our assembled genome, we further sequenced the genomic DNA using the Pacbio RSII platform (Pacific Biosciences, CA, United States). A 40-kb SMRTbell DNA library was prepared to perform the Pacbio sequencing.

### *De novo* Assembly and Annotation of the *H. oryzae* Mitogenome

Illumina PCR adapter reads and low-quality reads from the paired-end reads were filtered using custom scripts. Clean reads were obtained after the quality control step. The mitogenome of *H. oryzae* was assembled by CANU v1.6 (Koren et al., 2017) using the Pacbio long reads. The assembled contigs were further polished using the paired-end Illumina reads with Pilon v1.22 (Walker et al., 2014). The obtained *H. oryzae* complete mitogenome was annotated according to the methods previously described (Li et al.,2020b,c). Briefly, the complete mitogenome of *H. oryzae* was firstly annotated based on the results of MITOS (Bernt et al., 2013) and MFannot (Valach et al., 2014). At this step, PCGs, ribosomal RNA (rRNA) genes, and tRNA genes were initially annotated. Open reading frames (ORFs) were modified or predicted with the NCBI Open Reading Frame Finder [ORFs less than 100 amino acids (aa) were excluded] (Coordinators, 2017) and annotated with BLASTp searches against the NCBI non-redundant protein sequence database (Bleasby and Wootton, 1990). tRNA genes were also predicted with tRNAscan-SE v1.3.1 (Lowe and Chan, 2016). The graphical map of the complete mitogenome was drawn with OGDraw v1.2 (Lohse et al., 2013).

### Sequence Analysis

The base composition of the *H. oryzae* mitogenome was analyzed using DNASTAR Lasergene v7.1^2^ software. Strand asymmetry of the *H. oryzae* mitogenome was assessed using the following formulas: AT skew = [A − T]/[A + T] and GC skew = [G − C]/[G + C] (Wang et al., 2017). The non-synonymous substitution rate (Ka) and the synonymous substitution rate (Ks) for the core PCGs in the four Tremellales mitogenomes were calculated using DnaSP v6.10.01 (Rozas et al., 2017). We used MEGA v6.06 (Caspermeyer, 2016) to calculate the overall mean genetic distances between each pair of the 15 core PCGs (*atp6*, *atp8*, *atp9*, *cob*, *cox1*, *cox2*, *cox3*, *nad1*, *nad2*, *nad3*, *nad4*, *nad4L*, *nad5*, *nad6*, and *rps3*) using the Kimura-2-parameter (K2P) model. The genome synteny of the closely related mitogenomes was analyzed using Mauve v2.4.0 (Darling et al., 2004). The introns of the *cox1* gene in 33 basidiomycete species were classified into different position classes (Pcls) and named according to previously described methods (Zhang and Zhang, 2019).

### Phylogenetic Analysis

In order to investigate the phylogenetic status of *H. oryzae* among the Basidiomycota phylum, we constructed a phylogenetic tree of 33 species based on the combined mitochondrial gene set (15 core PCGs + two rRNA genes) (Li et al., 2018d). The individual mitochondrial gene was first aligned using MAFFT v7.037 (Katoh et al., 2019). Then, these alignments were concatenated in SequenceMatrix v1.7.8 (Vaidya et al., 2011). In order to detect potential phylogenetic conflicts between different genes, we carried out a preliminary partition homogeneity test. Phylogenetic trees were constructed using both Bayesian inference (BI) and maximum likelihood (ML) methods. Best-fit models of evolution and partitioning schemes for the gene set were determined according to PartitionFinder 2.1.1 (Lanfear et al., 2017). BI analysis was performed with MrBayes v3.2.6 (Ronquist et al., 2012). The RAxML v 8.0.0 software (Stamatakis, 2014) was used for ML analysis.

### Data Availability Statement

The newly sequenced *H. oryzae* mitogenome was deposited in the GenBank database under the following Accession No.: MH732752.

## Results

### Protein-Coding Genes, RNA Genes, and Codon Analysis in the *H. oryzae* Mitogenome

**FIGURE 1:**
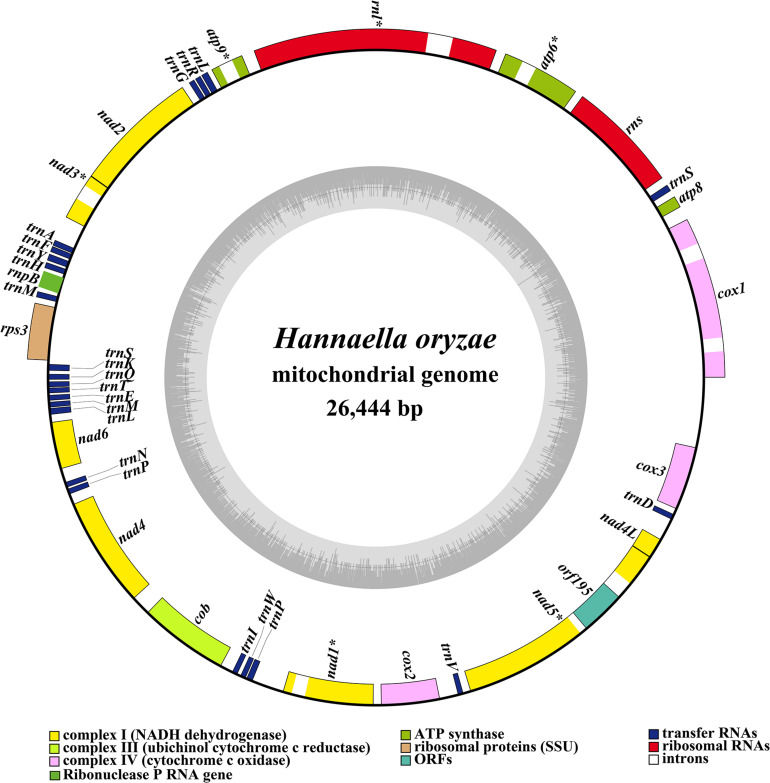
Circular map of the mitochondrial genome of *Hannaella oryzae*. Genes are represented by *different colored blocks*. *Colored blocks outside each ring* indicate that the genes are on the direct strand, while *colored blocks within the ring* indicate that the genes are located on the reverse strand. The *inner grayscale bar graph* shows the GC content of the mitochondrial sequences. The *circle inside the GC content graph* marks the 50% threshold. The graphical map of the complete mitogenome was drawn with OGDraw v1.2 (Lohse et al., 2013).

The complete mitochondrial genome of *H. oryzae* was assembled into a circular DNA molecule with a total size of 26,444 bp (Figure 1). The GC content of the *H. oryzae* mitogenome was 38.98% (Supplementary Table 1). Both the GC skew and the AT skew of the *H. oryzae* mitochondrial genome were positive. Fifteen conserved PCGs were detected in the *H. oryzae* mitogenome, including 14 core PCGs for energy metabolism and one *rps3* gene (Supplementary Table 2). Eight introns were detected in the mitogenome of *H. oryzae* distributed in seven host genes, including *cox1*, *atp6*, *rnl*, *atp9*, *nad3*, *nad1*, and *nad5*. The *cox1* gene contained two introns, while the other six genes each contained one intron. All these introns belonged to group I, which could catalyze their own splicing. One intronic ORF (*orf195*) was found in the *H. oryzae* mitochondrial genome, which encoded LAGLIDADG homing endonuclease. Of the 42 genes detected in the *H. oryzae* mitogenome, 19 were on the direct strand and the other 23 were on the reverse strand.

The mitogenome of *H. oryzae* contained two rRNA genes, namely the small subunit ribosomal RNA (*rns*) and the large subunit ribosomal RNA (*rnl*) (Supplementary Table 2). Twenty-three tRNA genes were detected in the *H. oryzae* mitogenome, encoding for 19 standard amino acids. The *H. oryzae* mitogenome lacked a tRNA gene that encoded for amino acid cysteine. Interestingly, we found that all four species from the order Tremellales lacked the tRNA gene to transfer cysteine. We detected about 40 bases of homology to Cys-tRNAs in the four mitogenomes. In addition, the *H. oryzae* mitogenome contained two tRNAs with the same anticodons coding for methionine and proline (Supplementary Figure 1). However, these two tRNAs varied in length and base composition, indicating frequent gene mutations in tRNA genes. The *H. oryzae* mitogenome also contained two tRNAs with different anticodons that coded for leucine and serine. The mitogenome of *H. oryzae* lost a tRNA gene with different anticodons that coded for arginine. The length of individual tRNAs ranged from 71 to 85 bp, which was mainly due to the variations in length of the extra arm. A ribonuclease P RNA gene (*rnpB*) was found in the *H. oryzae* mitogenome with a length of 211 bp.

The sizes of the five mitogenomes varied greatly, ranging from 24,874 to 35,058 bp. Correlation analysis showed that the intronic regions had the greatest impact on the size variations of Tremellales and Trichosporonales mitogenomes, with Pearson’s correlation coefficient of 0.907 (*P* < 0.05; Supplementary Figure 2). The results suggested that the intronic region was the main factor promoting the size variations of mitogenomes in Tremellales and Trichosporonales.

### Intron Evolution of *H. oryzae* Mitogenome

**FIGURE 2:**
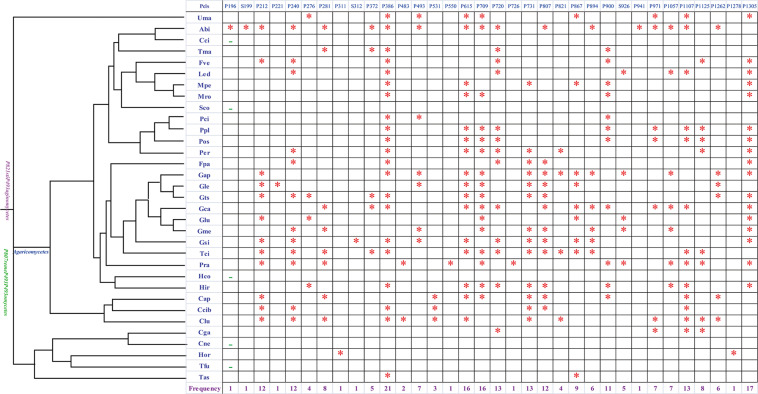
Position class (Pcl) information of *cox1* introns in 33 basidiomycete species. The phylogenetic tree on the *left* was constructed using the Bayesian inference (BI) and maximum likelihood (ML) methods based on a combined mitochondrial gene set (Bayesian posterior probabilities and bootstrap support values are shown in Figure 5). Pcls are named according to previously described methods (Zhang and Zhang, 2019). Species IDs for the mitogenomes used in the intron analysis are provided in Supplementary Table 4. *Asterisk* indicates the presence of the Pcl in the species and an *en dash* indicates the absence of any Pcl in the species. The *number at the bottom* indicates the frequency of occurrence of the Pcl in the 33 basidiomycete species.

Introns were considered as the main factors leading to variations of the mitogenome size in Tremellales, Trichosporonales, and other basidiomycetes. So the dynamic changes of the introns in *H. oryzae* and other basidiomycetes were analyzed in depth in the present study. The *cox1* gene was found to be the largest host gene of introns in basidiomycetes (Ferandon et al., 2010). Introns in the *cox1* gene could be divided into different Pcls according to the insertion sites in the coding region. The introns from the same Pcls were considered homologous introns with high sequence similarities (Ferandon et al., 2010). Introns belonging to different Pcls were considered to be non-homologous and contained low sequence similarities. In the present study, a total of 246 introns of the *cox1* gene were detected in the 33 basidiomycete species we tested, seven of which belonged to group II and the others belonged to group I (Figure 2). The 239 group I introns could be classified into 31 Pcls, 26 of which were reported in previous studies (Ferandon et al., 2010), while the other five were novel Pcls found in the 33 basidiomycetes. Among these Pcls, Pcl P386 was the most widely distributed, distributed in 21 of 33 basidiomycete species. Pcls P615 and P709 were also common Pcls in basidiomycetes, both of which existed in 16 of the 33 basidiomycete species. Pcls P196, P221, and P941 were found only in one of the 33 basidiomycete species, which were considered as rare introns in basidiomycetes. Eight introns were detected in the *cox1* gene of five species from Tremellales and Trichosporonales. *Cryptococcus neoformans* and *Tremella fuciformis* lost introns in the *cox1* gene, while the *cox1* gene of *Cryptococcus gattii* contained the most introns among Tremellales and Trichosporonales, indicating that species in Tremellales and Trichosporonales had experienced intron loss/gain events during evolution. In addition, *H. oryzae* was found to contain two novel intron Pcls at sites 103 and 425 aa (Supplementary Table 3). No homologous introns were found in the four closely related species and other basidiomycete species, indicating that *H. oryzae* had undergone a unique intron origin.

### Variations, Genetic Distance, and Evolution Rate of Core Genes

Of the 15 core PCGs detected in the four Tremellales mitogenomes, 12 PCGs were found to vary in length among the four species, except for *atp8*, *atp9*, and *nad4L* (Supplementary Figure 3). Nine genes of the *H. oryzae* mitogenome had unique lengths among the four Tremellales mitogenomes, including the *cob*, *cox1*, *cox3*, *nad2*, *nad3*, *nad4*, *nad5*, *nad6*, and *rps3* genes. The GC contents of the core PCGs in the four Tremellales mitogenomes also varied, indicating frequent base variations in the core PCGs of the Tremellales mitogenomes. Among the 15 core PCGs detected, *atp9* contained the highest GC content in all four Tremellales mitogenomes. The AT and GC skews of most of the core PCGs were negative in the four mitogenomes. The GC skews of the 15 core PCGs in the *H. oryzae* mitogenome were negative. The *rps3* gene contained a positive AT skew in the *H. oryzae* mitogenome.

Of the 15 core PCGs detected, the *rps3* gene had the highest mean K2P genetic distance among the four Tremellales mitogenomes, followed by *nad3* (Supplementary Figure 4). The mean genetic distance of *atp9* in the four mitogenomes was the smallest among the 15 core PCGs, indicating that this gene was highly conserved across the mitogenomes. *nad4L* contained the highest mean Ks among the 15 core PCGs detected, while *atp9* had the lowest rate. The highest Ka was observed in the *rps3* gene, while *nad4L* exhibited the lowest Ka value among the 15 PCGs detected. The Ka/Ks values for all 15 PCGs were less than 1, indicating that these genes were subject to purifying selection.

### Comparative Genome Analysis

The mitogenome size of *H. oryzae* was the second smallest among all published Basidiomycota mitochondrial genomes (see text footnote 1), only higher than that of the basidiomycete yeast *C. neoformans* (Yan et al., 2018; Supplementary Table 1). The mitogenome size of *H. oryzae* was smaller than that of mushroom-forming species, such as *Pleurotus* spp. (Li et al., 2018a), *Ganoderma* spp. (Li et al., 2018d, 2019c), *Coprinopsis cinereal* (Stajich et al., 2010), and *Agaricus bisporus* (Ferandon et al., 2013), and its closely related species, *T. fuciformis* (MF422647); smaller than that of the ectomycorrhizal fungi *Tricholoma matsutake* (Yoon et al., 2016), *Lactarius* spp. (Li et al., 2019b), *Russula* spp.(Li et al., 2018c), and *Cantharellus* spp. (Li et al., 2018b); and also smaller than that of the basidiomycete yeast *C. gattii* (Yadav et al., 2018). The GC content of the *H. oryzae* mitogenome was relatively high (38.98%), which was only lower than that of *Rhodotorula mucilaginosa* (40.43%) (Gan et al., 2017), among all the published Basidiomycota mitochondrial genomes detected. There were seven, seven, two, and four introns detected in the mitogenomes of *T. fuciformis*, *C. gattii*, *C. neoformans*, and *Trichosporon asahii*, which contained five, six, one, and four introns, respectively. However, eight introns were detected in the mitogenome of *H. oryzae*, but only one of them contained intronic ORF, suggesting that the mitogenome introns of *H. oryzae* were undergoing constriction.

### Gene Rearrangement Analysis

**FIGURE 3:**
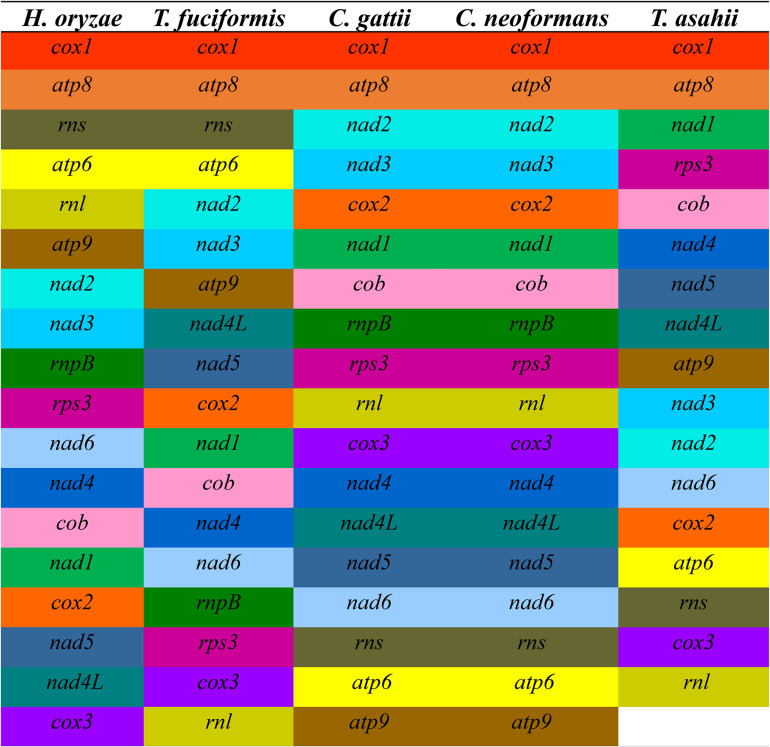
Comparison of the conserved gene order among the mitochondrial genomes of the five closely related species in Tremellales and Trichosporonales, including *Cryptococcus gattii*, *Cryptococcus neoformans*, *Hannaella oryzae*, *Tremella fuciformis*, and *Trichosporon asahii*. Fifteen core protein-coding genes (PCGs) and two rRNA genes were included in this analysis. Genes are represented by *different colored blocks*.

The gene arrangement in the mitogenomes of five closely related species in Tremellales and Trichosporonales was highly variable (Figure 3). Of the 18 genes detected in the five closely related species, including the 15 core PCGs, two rRNA genes, and one *rnpB* gene, the relative positions of the 16 genes varied among the five mitogenomes. The gene arrangements of *C. gattii* and *C. neoformans* were highly conserved between the two species. However, at the class level, the arrangement of the mitochondrial genes was highly variable. *H. oryzae* contained a unique gene arrangement in the order Tremellales, indicating that gene rearrangements have occurred during the evolution of *H. oryzae*, involving protein-encoded genes, rRNA genes, and the *rnpB* gene.

**FIGURE 4:**
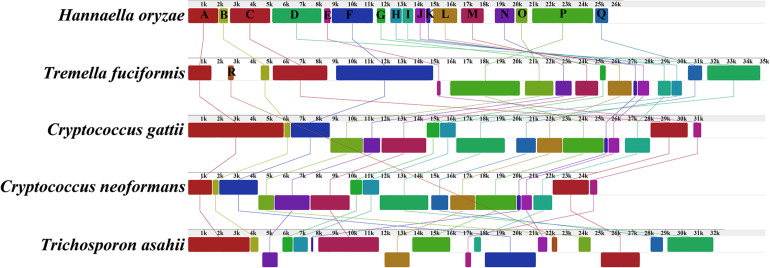
Collinearity analysis of the mitochondrial genomes of the five closely related species in Tremellales and Trichosporonales, including *Cryptococcus gattii*, *Cryptococcus neoformans*, *Hannaella oryzae*, *Tremella fuciformis*, and *Trichosporon asahii*. Eighteen homologous regions were detected among the five mitogenomes. The sizes and the relative positions of the homologous regions varied among the mitogenomes. The genome synteny of the closely related mitogenomes was analyzed using Mauve v2.4.0 (Darling et al., 2004).

The genome collinearity analysis showed that the five mitogenomes of Tremellales and Trichosporonales could be divided into 18 homologous regions (Figure 4). The relative positions of these homologous regions were highly variable among the five Tremellales mitogenomes. Out of the 18 homologous regions, the relative positions of 17 homologous regions varied among the five mitogenomes. The relative positions of the homologous regions were identical between *C. gattii* and *C. neoformans*.

### Phylogenetic Analysis

**FIGURE 5:**
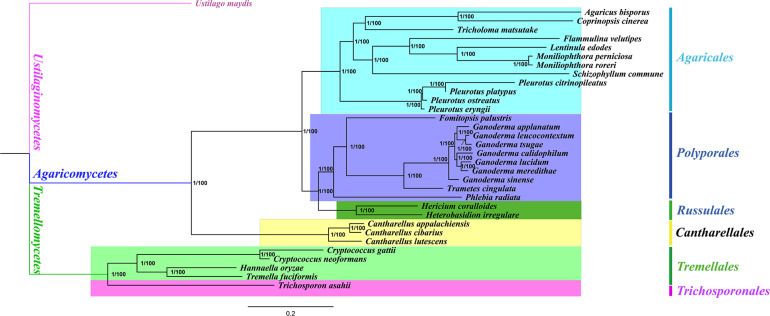
Molecular phylogeny of 33 Basidiomycota species based on both Bayesian inference (BI) and maximum likelihood (ML) analyses of 15 core protein-coding genes and two rRNA genes. Support values are Bayesian posterior probabilities (BPP; *before the slash*) and bootstrap (BS; *after the slash*). The species and the NCBI accession numbers for the genomes used in the phylogenetic analysis are provided in Supplementary Table 4.

We obtained identical and well-supported tree topologies using both BI and ML methods based on the combined mitochondrial gene set (15 core PCGs + two rRNA genes) (Figure 5). All major clades of the trees were well supported [Bayesian posterior probability (BPP) = 1.00, bootstrap (BS) = 100]. Based on the phylogenetic analyses, the 33 Basidiomycota species could be divided into seven major clades corresponding to the orders Ustilaginales, Agaricales, Polyporales, Russulales, Cantharellales, Tremellales, and Trichosporonales (Supplementary Table 4). The four species from Tremellales could be divided into two groups: one group was composed of two species in the *Cryptococcus* genus and the second group was composed of *H. oryzae* and *T. fuciformis*. *H. oryzae* was identified as a sister species to *T. fuciformis*. The results showed that the combined mitochondrial gene set was suitable as a reliable molecular marker for the analysis of the phylogenetic relationships among Basidiomycota species.

## Discussion

As the “second genome” of eukaryotes, the mitochondrial genome plays an important role in regulating the growth and development, energy metabolism, aging, and stress resistance of eukaryotes (Bhargava and Schnellmann, 2017; Scheede-Bergdahl and Bergdahl, 2017). With the development of the next-generation sequencing technology, more and more mitochondrial genomes have been obtained, which has promoted our understanding of the origin, evolution, and taxonomy of eukaryotes (du Toit et al., 2017; Yang et al., 2018; Zhang et al., 2018). However, compared with animals, the mitochondrial genomes of fungi were less studied, especially that of Basidiomycota. It is estimated that there are more than 30,000 species of Basidiomycota in nature. However, up to now, less than 100 complete mitochondrial genomes of Basidiomycota are available in the public database (see text footnote 1). Only four mitochondrial genomes of basidiomycete yeasts were reported. This report of the mitochondrial genome of the important phylloplane yeast *H. oryzae* will broaden our understanding of basidiomycete mitochondrial genomes. The mitochondrial genome size of *H. oryzae* is the second smallest among all published Basidiomycota mitochondrial genomes, smaller than those of many mushroom-forming fungi (Stajich et al., 2010; Yadav et al., 2018), ectomycorrhizal fungi (Li et al., 2018b, 2020c), and that of its related species, *T. fuciformis*. However, the GC content of the *H. oryzae* mitogenome is the second highest among all published Basidiomycota mitochondrial genomes, indicating its unique mitogenome characteristics. Comparative mitogenomic analysis showed that the intron region had the greatest effect on the variations of the mitochondrial genome size of Tremellales, followed by the intergenic region and the protein-coding region, which was consistent with previous studies (Li et al., 2018b). Introns of the Tremellales *cox1* gene were found to have undergone intron loss/gain events, which resulted in the size and organization variations of the Tremellales mitogenomes. In addition, the *cox1* gene of *H. oryzae* contained two novel introns, which showed different origins from the known introns in basidiomycetes. Interestingly, we did not find any intron homologous to the introns of the *H. oryzae cox1* gene in the NCBI database by BlastN search. More mitochondrial genomes need to be obtained to reveal the origin of the introns in *H. oryzae* or other basidiomycete species. Mitochondrial genes of *H. oryzae* were found on both strands as compared with the ascomycete mitochondrial genes, which are usually on the same strand (Aguileta et al., 2014).

It was reported that the mitochondrial genome of eukaryotes was obtained from a common alpha-proteobacterium ancestor (Lang et al., 1999). As evolution progresses, most mitochondrial genes were transferred to the nuclear genome, which was considered to have many advantages (Adams et al., 2002; Adams and Palmer, 2003). However, most fungi retain 14 conserved protein-coding genes for energy metabolism and one *rps3* gene, which were called the core PCGs of the fungal mitogenome (Ye et al., 2020). In this study, these core PCGs were found to have high variation rates in the length and base composition even among the closely related species. Nine genes of *H. oryzae* were found to have unique lengths in the five closely related species, indicating the unique evolutionary characteristics of *H. oryzae*. Of all core PCGs, *atp9* was found the most conserved among the five closely related species we studied. The genetic distance of the *rps3* gene was the largest among the five closely related species we examined, which was consistent with the report in other literature (Li et al., 2018b, 2020c). Interestingly, we found that the *atp8* gene in Tremellomycetes and Ustilaginomycetes had 12 nucleotides missing compared with that in Agaricomycetes species; the effect of the base variation of PCGs on the energy metabolism of species needs to be examined. In addition, we found that the core PCGs of the five closely related species in Tremellales and Trichosporonales were subject to purifying selection.

The arrangement of mitochondrial genes can serve as an important reference for revealing the evolutionary position of species (Li et al., 2018a; Zheng et al., 2018). Mitochondrial gene arrangement has been extensively studied in animals, and several models have been proposed to explain the rearrangement of mitochondrial genes in animals (Boore, 1999; Perseke et al., 2008). Compared with animals, the arrangement of fungal mitochondrial genes is highly variable (Hamari et al., 2001; Aguileta et al., 2014; Li et al., 2018a), and the gene rearrangement of the fungal mitochondrial genome has not been fully understood. Previous studies have shown that the accumulation of repetitive sequences in the fungal mitogenome has caused the fungal mitogenome rearrangement (Aguileta et al., 2014). In the present study, we found that the arrangement of mitochondrial genes was highly variable in the five closely related species, and the mitogenome of *H. oryzae* contained a unique gene order. In addition, the gene order varied at different taxonomic levels and is highly conserved between two species of the *Cryptococcus* genus. However, large-scale gene rearrangements have been observed at the class level, suggesting that Tremellales species undergo large-scale rearrangement events during evolution. Mitochondrial genes are also widely used as important molecular markers in the study of evolution, phylogeny, and population genetics (Bronstein and Kroh, 2018; Dai et al., 2018; Doyle et al., 2018; Li et al., 2020a). In this study, we divided 33 Basidiomycota species into seven clades based on a phylogenetic tree with high support for 15 core PCGs and two rRNA genes. Because there are few morphological features for fungi to be identified, this leads to confusion in fungal taxonomy and affects the phylogenetic research and utilization of fungi. The introduction of the mitochondrial genome will promote the understanding of fungal taxonomy and genetic evolution and can be used as a reliable tool for the analysis of fungal phylogeny (Li et al., 2018c, 2019a). More fungal mitochondrial genomes need to be uncovered to reconstruct the phylogenetic tree of fungi and to clarify the phylogenetic status of the Basidiomycota species.

## Data Availability Statement

The datasets presented in this study can be found in online repositories. The names of the repository/repositories and accession number(s) can be found in the article/Supplementary Material.

## Author Contributions

QL and WH conceived and designed experiments. QL, LL, HF, WT, ZB, CX, and XW analyzed the data. QL, YQ, and WH wrote and revised the manuscript. All authors contributed to the article and approved the submitted version.

## Conflict of Interest

The authors declare that the research was conducted in the absence of any commercial or financial relationships that could be construed as a potential conflict of interest.

## Footnotes

1 https://www.ncbi.nlm.nih.gov/genome/browse#!/organelles/

2 http://www.dnastar.com/

## Supplementary Material

Supplementary Figure 1 Putative secondary structures of the 23 tRNA genes identified in the mitogenome of *H. oryzae*. All genes are shown in order of occurrence in the mitogenome of *H. oryzae*, starting from *trnS*.

Supplementary Figure 2 Pearson correlation analyses between mitochondrial genome sizes and different mitochondrial components of five closely related species in Tremellales and Trichosporonales.

Supplementary Figure 3 Variation in the length and base composition of each of 15 core protein-coding genes (PCGs) among the mitochondrial genomes of the four closely related species in Tremellales. **(A)** PCG length variation; **(B)** GC content of the PCGs; **(C)** AT skew; **(D)** GC skew.

Supplementary Figure 4 Genetic analysis of 15 core protein-coding genes conserved in the four closely related species in Tremellales. K2P, the Kimura-2-parameter distance; Ka, the mean number of non-synonymous substitutions per non-synonymous site; Ks, the mean number of synonymous substitutions per synonymous site.
