# Supplementary figures and images for: Characterization of the Complete Mitochondrial Genome of Basidiomycete Yeast Hannaella oryzae: Intron Evolution, Gene Rearrangement, and Its Phylogeny
Source: Front Microbiol. 2021 May 28;12:646567. doi: 10.3389/fmicb.2021.646567 (PMC8193148; doi:10.3389/fmicb.2021.646567)

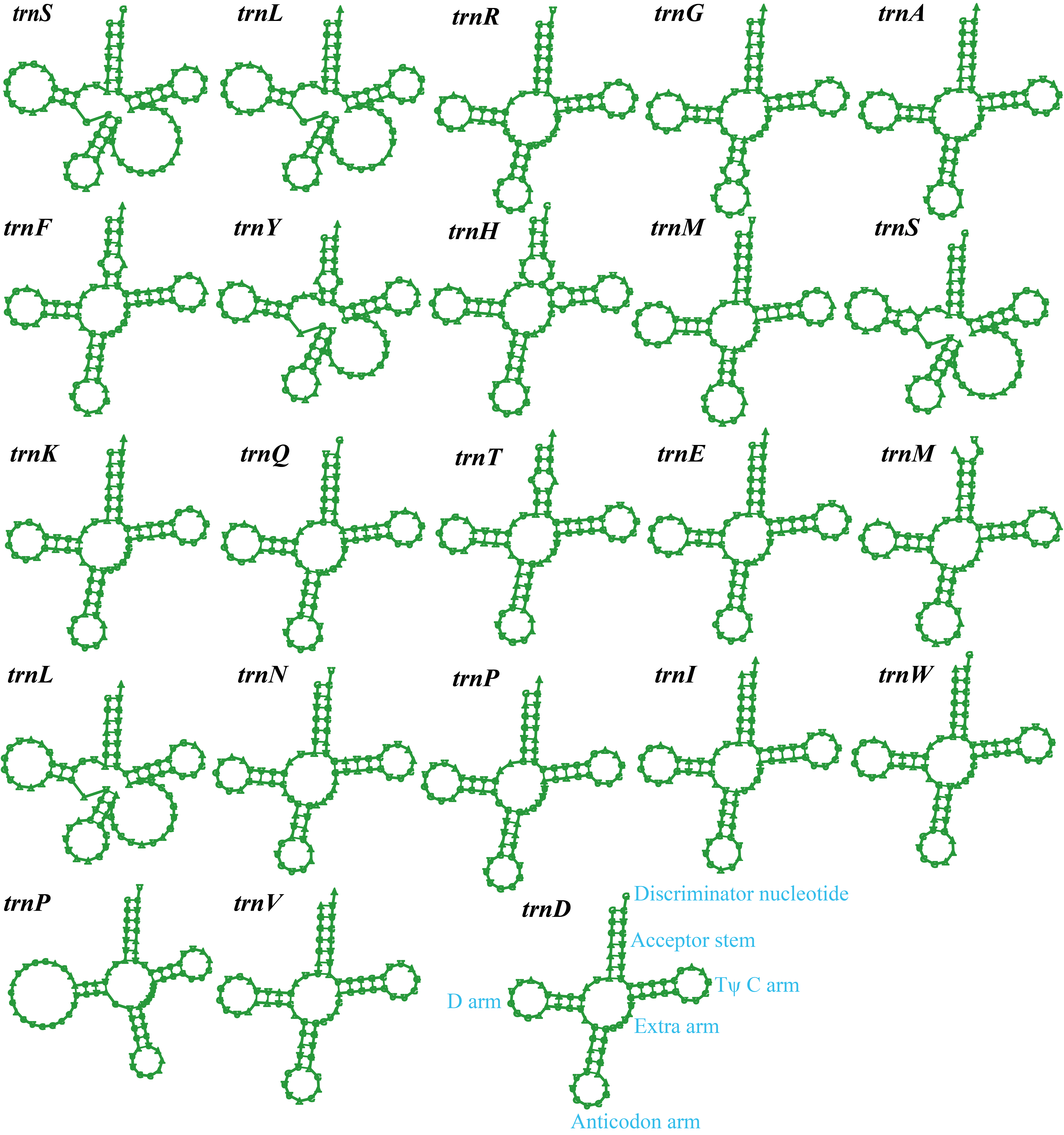

Supplement: Supplementary Figure 1 — Putative secondary structures of the 23 tRNA genes identified in the mitogenome of H. oryzae. All genes are shown in order of occurrence in the mitogenome of H. oryzae, starting from trnS. [file Image_1.TIF]

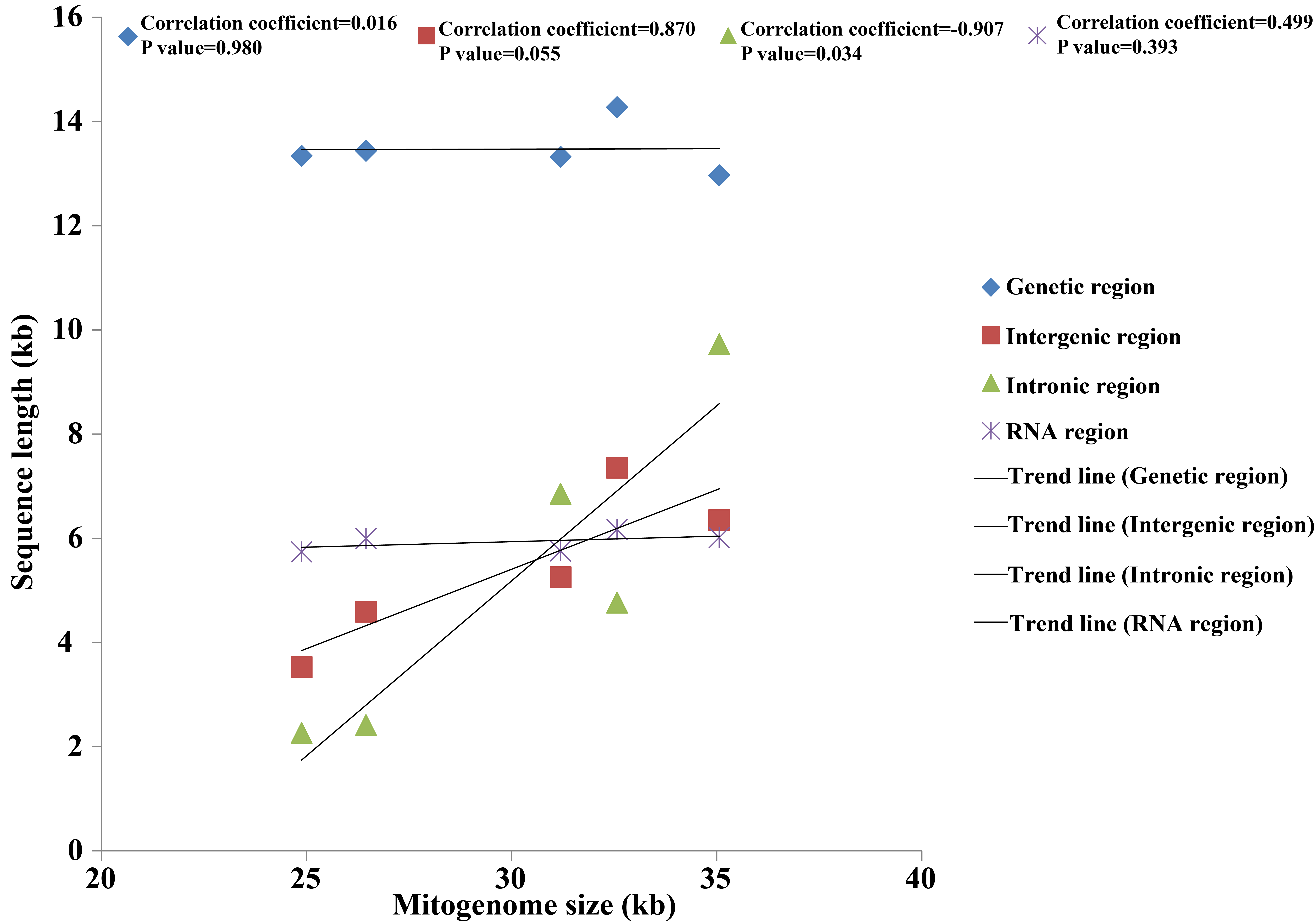

Supplement: Supplementary Figure 2 — Pearson correlation analyses between mitochondrial genome sizes and different mitochondrial components of five closely related species in Tremellales and Trichosporonales. [file Image_2.TIF]

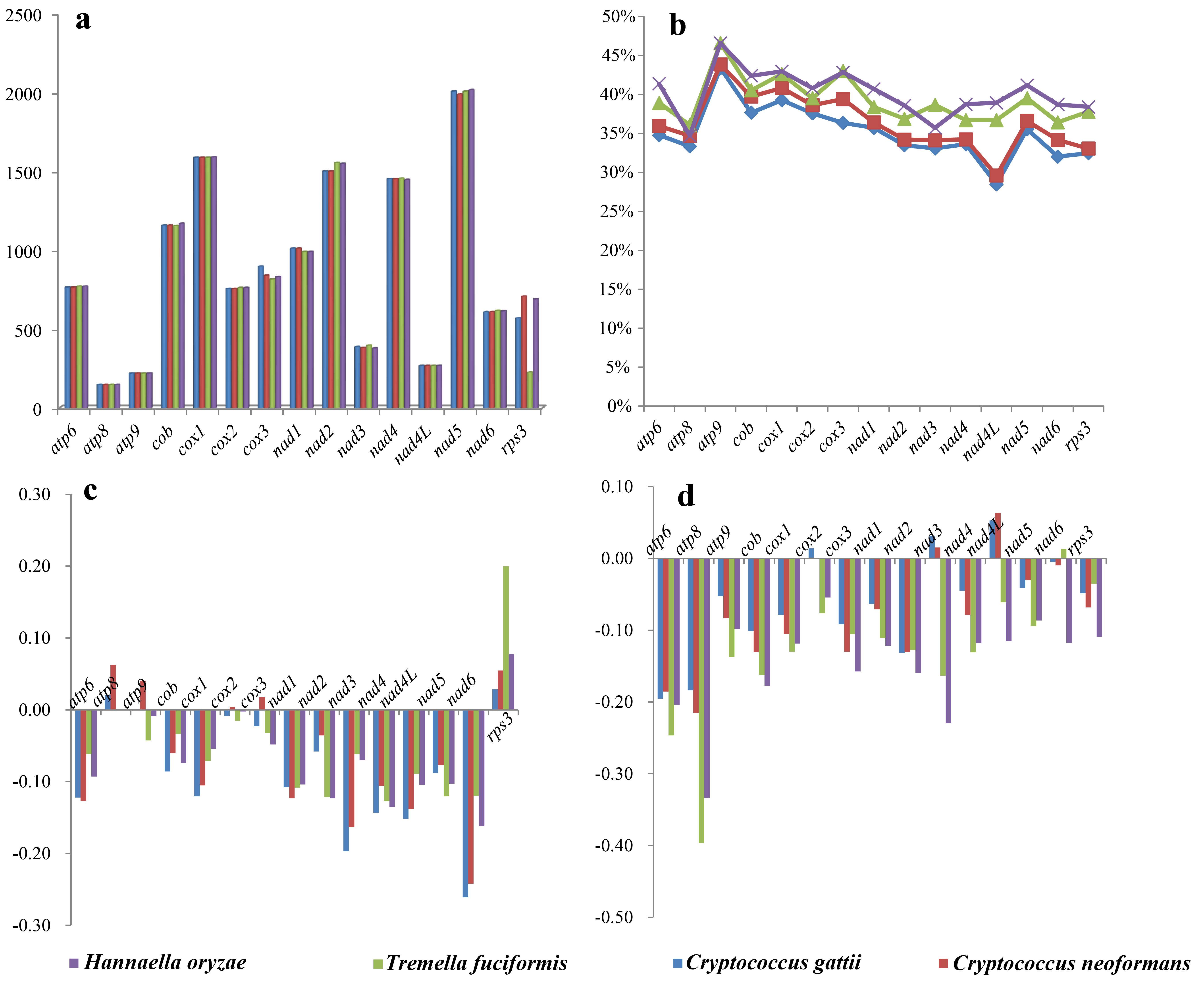

Supplement: Supplementary Figure 3 — Variation in the length and base composition of each of 15 core protein-coding genes (PCGs) among the mitochondrial genomes of the four closely related species in Tremellales. (A) PCG length variation; (B) GC content of the PCGs; (C) AT skew; (D) GC skew. [file Image_3.TIF]

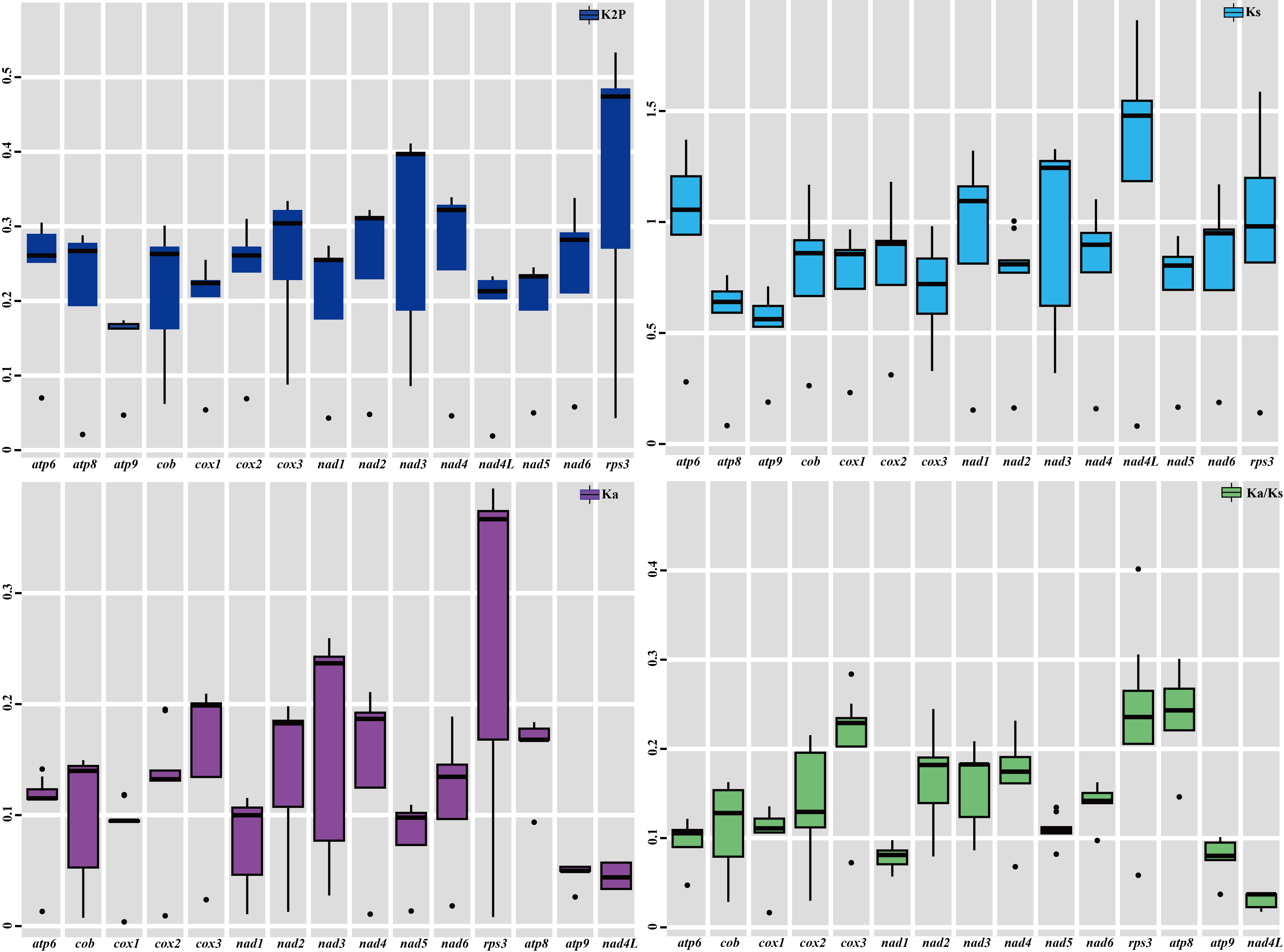

Supplement: Supplementary Figure 4 — Genetic analysis of 15 core protein-coding genes conserved in the four closely related species in Tremellales. K2P, the Kimura-2-parameter distance; Ka, the mean number of non-synonymous substitutions per non-synonymous site; Ks, the mean number of synonymous substitutions per synonymous site. [file Image_4.TIF]
